# Improving Access to Surgery Through Surgical Team Mentoring – Policy Lessons From Group Model Building With Local Stakeholders in Malawi

**DOI:** 10.34172/ijhpm.2021.78

**Published:** 2021-08-03

**Authors:** Henk Broekhuizen, Martilord Ifeanyichi, Gerald Mwapasa, Chiara Pittalis, Patrick Noah, Nyengo Mkandawire, Eric Borgstein, Ruairí Brugha, Jakub Gajewski, Leon Bijlmakers

**Affiliations:** ^1^Radboud Institute for Health Sciences, Radboud University Medical Centre, Nijmegen, The Netherlands.; ^2^Department of Health and Society, Wageningen University and Research, Wageningen, The Netherlands.; ^3^College of Medicine, Blantyre, Malawi.; ^4^Institute of Global Surgery, Royal College of Surgeons in Ireland, Dublin, Ireland.; ^5^Department of Epidemiology and Public Health Medicine, Royal College of Surgeons in Ireland, Dublin, Ireland.

**Keywords:** Global Surgery, Access, Surgical Mentoring, Sustainability, Group Model Building, Malawi

## Abstract

**Background:** There is much scope to empower district hospital (DH) surgical teams in low- and middle-income countries to undertake a wider range and a larger number of surgical procedures so as to make surgery more accessible to rural populations and decrease the number of unnecessary referrals to central hospitals (CHs). For surgical team mentoring in the form of field visits to be undertaken as a routine activity, it needs to be embedded in the local context. This paper explores the complex dimensions of implementing surgical team mentoring in Malawi by identifying stakeholder-sourced scenarios that fit with, among others, national policy and regulations, incentives to perform surgery, career opportunities, competing priorities, alternatives for performing surgery locally and the proximity and role of referral hospitals.

**Methods:** A mixed methods approach was used which combined stakeholder input – obtained through two group model building (GMB) workshops and further consultations with local stakeholders and SURG-Africa project staff – and dynamic modeling to explore policy options for sustaining and rolling out surgical team mentoring. Sensitivity analyses were also performed.

**Results:** Each of the two GMB workshops resulted in a causal loop diagram (CLD) with an array of factors and feedback loops describing the complexity of surgical team mentoring. Six implementation scenarios were defined to perform such mentoring. For each the resource requirements were identified for the institutions involved – notably DHs, CHs and the party that would finance the required mentoring trips – along with the potential for scaling up surgery at DHs under severe financial constraints.

**Conclusion:** To sustain surgical mentoring, it is important that an approach of continued communication, monitoring, and (re-)evaluation is taken. In addition, an output- or performance-based financing scheme for DHs is required to incentivize them to scale up surgery.

## Background

**Key Messages**

** Implications for policy makers**

- Mentoring of district hospital (DH)-level surgical teams is best done through a staggered, nation-wide roll-out of field trips, and a dedicated senior coordinator who is in regular contact with both central hospital (CH) and DH.
- It is critical to monitor surgical performance during periodic meetings with mentors and mentees, and to assess the acceptability and effectiveness of surgical team mentoring.
- Inclusion of in-service training and mentoring activities in the key performance indicators of both DHs and surgical specialists would improve their motivation to scale up safe surgery for rural populations.
- A mechanism of output- or performance-based financing of surgery is required to scale-up surgery at DHs and avoid unnecessary surgical referrals to higher level hospitals.

** Implications for the public**

Scaling up surgery at district hospitals (DHs) in order to make safe surgery more accessible for rural populations requires mentoring of DH surgical teams through periodic field visits by teams of senior surgical staff who are usually based at central hospitals (CHs). For such surgical team mentoring to be effective and carried out efficiently, there is need to (*i*) create a focal point with a dedicated national coordinator, (*ii*) routinely monitor DHs’ surgical performance, (*iii*) adequately reward both the mentors and the mentees, and (*iv*) introduce a financing mechanism through which DHs are financially compensated if they perform more surgery.

Worldwide, millions of people lack access to safe surgery.^1^ A large percentage of these live in low- and middle-income countries in sub-Saharan Africa. One of the main barriers to access is the general shortage of surgical personnel: surgeons, anesthetists, obstetricians, and theatre nurses. Such cadres in sub-Sahara Africa, especially the specialists, are often concentrated in urban areas where remuneration and living conditions are better. The people who are not able to access surgery are those living in rural areas. Here, non-specialists are usually the ones performing surgery at the local district hospitals (DHs), as part of DH-level surgical teams that further comprise anesthesia providers, theatre nurses and support staff.^2^ Non-specialists face a multitude of barriers to providing surgery, such as shortages of supplies, poor infrastructure, regulatory ambiguities, and lack of training and supervision,^3^ which poses challenges to their retention for work in rural areas.^4^ In Malawi, a country with 18 million inhabitants of whom 84% live in rural areas, there are only 42 surgical specialists, mostly working in the central hospitals (CHs) of Blantyre and Lilongwe. Most (surgical) care at Malawian DHs is provided by a cadre of non-physician clinicians, called clinical officers in Malawi.^5^ The European Union-funded SURG-Africa project was designed to demonstrate an effective way to improve district surgical capacity using locally available resources. It has implemented and evaluated surgical mentoring and supervision between 2018 and 2020.^6^ This intervention consisted of two complementary parts: periodic mentoring trips of DH surgical teams by specialists from CHs; and a managed remote surgical consultation network based on WhatsApp.^7^ The rationale behind the intervention was that surgical teams at DHs would be empowered in terms of professional knowledge, skills and confidence to undertake a wider range and a larger number of surgical procedures. Through this two-pronged intervention it was expected that surgery would become more accessible to rural populations and that the number of unnecessary referrals to CHs would decrease.

 Although the managed clinical network continues, the mentoring trips funded through SURG-Africa ended in early 2020, which coincided with the onset of the coronavirus disease 2019 (COVID-19) pandemic. The sustainability of this intervention’s effects hinges on the willingness and ability of the Malawian healthcare system to continue surgical mentoring once the Ministry of Health (MoH) approves the resumption of field visits. Such a continuation will depend on two conditions.^8^ Firstly, the intervention (its components, implementing parties, beneficiaries, products/outputs, health benefits/outcomes) needs to fit with the needs and features of the local setting (staffing situation, division of labor, work schedules, organizational culture). In SURG-Africa, ensuring a good fit was addressed through a participatory action research stage, prior to the start and during implementation of the mentoring intervention.^9^ It entailed the design and shaping of the mentoring model, along with periodic reviews and adaptations of the intervention, where needed, based on workshops with mentors and mentees. Secondly there needs to be a fit between the intervention and the broader ecological context (policy, regulations, incentives, career opportunities, competing priorities, alternatives for performing surgery locally, proximity of referral hospitals). Such a fit is important for a sustainable uptake and scaling up of the intervention in routine practice. The aim of this paper is to explore stakeholder-sourced scenarios that would optimize the second fit. The central research question was: what would be the most suitable scenario for a continuation and possible further expansion of surgical team mentoring in Malawi? Based on the findings from two group model building (GMB) workshops and further consultations with local stakeholders and project staff we defined six implementation scenarios. For each scenario we explore future resource requirements for the institutions involved: DHs, CHs, and the party or stakeholder that would finance the mentoring trips. We also explore likely ‘soft’ consequences of scenarios, based primarily on GMB findings. From these we formulate policy recommendations.

## Methods

###  Group Model Building Workshops

 Two GMB workshops were held in September 2019: one in Nsanje district, the southern-most district of Malawi, selected on purpose because of its remoteness (more than 180 km away from Queen Elizabeth Central Hospital [QECH] in Blantyre) and particular interest in surgical mentoring; the other in Salima, the venue for a national-level workshop. The Nsanje workshop explored the requirements for performing district-level surgery and the possible consequences of scaling up surgical services locally, ie, Nsanje DH. A total of 24 participants drawn from Nsanje district council, the DH, and several nearby health centers participated in the workshop. Both clinicians and administrators participated. In Salima, the focus was on the dynamics of surgical mentoring, its sustainability in Southern region, where the SURG-Africa supported mentoring intervention was being implemented, and its replicability in the country’s two other regions. The total of 23 workshop participants included representatives from the Directorate of Clinical Services of the MoH, surgical mentors from two CHs and clinical staff from several DHs, most of which had received surgical mentoring visits. Among the participants several cadres (consultants, registrars, medical officers, clinical officers, nurses, anesthetists) were represented.

 Although the topic matter differed between the two workshops, the same general process was followed. Both workshops were facilitated by a moderator (author LB), a ‘model builder’ (conversant with Vensim software; HB), and a note taker (MI). Each workshop started with the facilitating team describing the purpose of the workshop and soliciting active participation and having an open mind for the experiences of fellow participants. A visual model was then developed in several rounds of variable elicitation, each starting with the nominal group technique.^10^ This method required participants to take some time to think by themselves and then write down on paper cards the factors affecting (in our case) surgical scale-up and sustainability of the mentoring. Going around the room, participants were asked to mention the factors they had identified. These were added to a causal loop diagram (CLD) that was projected on a screen in real-time so participants could immediately see their contributions and correct the facilitating team in case their input was misunderstood. After all factors were added to the screen, causal relationships between factors were discussed and added by the group. The model building was completed when no further factors or relationships were contributed, after which the group reviewed the whole diagram. Upon completion of the diagram in Salima, two sub-groups of participants discussed options for sustaining the intervention, guided by the following three questions: what are the conditions for sustaining the intervention, what could be done to ensure that these conditions are fulfilled, and which party/stakeholder is in the best position to do this?

###  Resource Requirement Projections: Model Descriptions

 We estimated the resource requirements of sustaining mentoring over time with a horizon of 5 years and counted in months with *t*_0_ = 0 being the first month of the SURG-Africa project (ie, March 2018); *t*_1_ being the end of SURG-Africa supported mentoring activities in March 2020 as per plan, which coincided with the suspension of all field trips in Malawi due to the COVID-19 pandemic; and *t*_2 _as the end of the 5 years horizon, in early 2023. We considered 3 main stakeholders: DHs, CHs, and the party that would take over financing of the mentoring trips from SURG-Africa. We included 23 government-owned DHs, denoted with *i* (full list in Supplementary file 1); and four CHs, denoted with *j*: Mzuzu hospital in Northern region; Kamuzu hospital in Lilongwe, Central region; and QECH in Blantyre and Zomba hospital (both in Southern region).

 The referrals that are sent from DH *i* to its referral hospital in month *t* was expressed as *r*_i_*(t)*. From previous studies we know that the intervention increases local team skills and confidence, which in turn decreases patient referrals to the CH.^7^ The effectiveness of the intervention (ie, mentoring trips and managed clinical network) is denoted with *x(t)* and is measured from 1 (no change in referrals) to 0 (all referrals avoided). The optimal effectiveness is unlikely to be 0 as appropriate referrals are essential in a functioning surgical system. We assume that a change in the number of referrals has two resource-related effects for DHs. Firstly, there will be savings as any avoided referral will reduce the DH’s expenditure on fuel, vehicle servicing, and travel allowances, with *c*_ri_ denoting the cost per referral from DH *i*. Secondly, with each avoided referral the DH may incur more costs for doing local surgery, with *c*_si_ the cost per major surgery at DH *i.* The costs per month *C*_i_*(t)* is thus:


(1) $$C_{i}\left(t\right)=\;x\left(t\right)r_{i}\left(t\right)\left(\gamma c_{si}+\;\alpha \beta c_{si}-\;c_{ri}\right)$$

Here, *γ *is the percentage of avoided referrals that is operated on at the DH (this does not need to be 1 as it is possible patients can be safely sent home, for example). Parameter *α* is the percentage of avoided referrals that is treated conservatively and thus does not receive surgery. Instead they are treated at the ward where the incurred costs are less than a full operation, ie, *βc*_si_ with *β* a number between 0 and 1. Mentoring is cost-saving from the DH perspective if *C*_i_*(t)* < 0.

 The resource implications of surgical mentoring trips for CHs are as follows. First of all, there is the reduction of referrals received from DHs and the associated reduction in bed occupancy in wards. To calculate referrals to each CH *j* (denoted as *R*_j_) we summed up all referrals based on the existing referral patterns in Malawi:


(2) $$R_{j}\left(t\right)=\sum_{i\in J}p_{ij}r_{i}\left(t\right)$$

Where *J* is the set of DHs that refer to CH *j* and *p*_ij_ is the percentage of referrals from DH *i* that go to CH *j*. The latter term was included to accommodate the fact that some DHs refer cases to multiple CHs. The total impact on bed occupancy, *o*_j_, was calculated by multiplying incoming referrals with the average length of stay of a surgical patients in a hospital *l*, which means that *o*_j_(*t*) = *Rj*(*t*)*l*. The second effect on CHs is the time spent on mentoring trips by specialists. To calculate this, we defined *f* as the number of mentoring trips undertaken per DH per year. We assume each mentoring trip takes two working days, as in the SURG-Africa model. Then the average number of days a CH would need to perform per month is:


(3) $$P_{j}=\sum_{i\in J`}\frac{2f}{12}$$

Here, J` is the set of DHs that is mentored by CH *j*. We assume all DHs in Southern region would receive mentoring visits from QECH, with no role in surgical mentoring for Zomba CH, as there is only one available mentor there. This means that Kamuzu CH and Mzuzu CH would mentor all DHs in their respective regions ( J=J` for Central and Northern region) and that DHs in Zomba catchment area would be visited by teams from Queen Elizabeth CH ( J≠J` for Southern region, as already happens in SURG-Africa). It is possible that some trips are cancelled, we denote this probability with *p*_cancel_. This makes the average number of days per CH per month.


(4) $$P_{j}=\left(1-p_{cancel}\right)\sum \frac{2f}{12}$$

To calculate the monthly requirements placed on individual mentoring specialists at a particular hospital we divided the total number of mentoring days needed in a month by the total trainer capacity of the hospital *kj*:


(5) $$d_{j}=\frac{P_{j}}{k_{j}}$$

Finally, there is a resource requirement for the party that would take over funding of the mentoring visits. There is a cost *c*_mi_ per mentoring trip to DH *i*. If each CH makes *f* visits to each DH in its region J` , the total monthly cost of the intervention is.


(6) $$\sum_{j}\left[P_{j}\sum_{i\in J`}c_{mi}\right]$$

When multiplied by 12 this becomes the yearly financial cost of mentoring *C*_m_.

###  Resource Requirement Projections: Parameter Estimates

 The SURG-Africa project produced data about referrals, cost of referrals, and cost of mentoring trips in the Southern region. However, there are two limitations to this data set for our aim of informing Malawi-wide policy. Firstly, we are assuming that the volume and cost of referrals and the cost of mentoring in the Central and Northern regions would not be much different from those in Southern region. Secondly, no data are available yet about the effects of the intervention after the visits stopped in early 2020. To meet our aims of exploring policy-relevant scenarios outside of the Southern region and beyond 2020, we fitted (log)linear models on best available data and filled in missing data with these. An overview of the sources for several key inputs can be found in Table 1.

**Table 1:** Overview of Sources for Selected Key Data and Parameters in the Resource Forecast Models

**Key Data and Parameters**		**Source**	
Cost	Cost of mentoring	Project data	Loglinear model
	Cost of referral	Ifeanyichi et al^13^ and loglinear model	Loglinear model
	Cost of providing surgery	Bijlmakers et al^14^ and imputation	Imputation
Referrals	During 2018	QECH registry and Ifeanyichi et al^13^	Maine et al^12^ and loglinear model
	After expiry of SURG-Africa	2018 data + model based on data from Pittalis et al^11^	
Parameters	% Of avoided referrals managed conservatively, *β*	10%, from Mwapasa et al^7^	
	% Of costs incurred at ward, *α*	38%, from Bijlmakers et al^14^	
	% Of avoided referrals operated locally, *γ*	25%, from Mwapasa et al^7^	
	% Of referrals that are surgical, *ρ*	40%, from Ifeanyichi et al^13^	
	Speed constant for mentoring effectiveness per trip, *λ*	-0.3, from model based on data from Pittalis et al^11^	

Abbreviation: QECH, Queen Elizabeth Central Hospital.

 From SURG-Africa data were available on the actual number of referrals from hospitals in the Southern region.^11^ For the number of referrals in the central region we used a study by Maine and colleagues.^12^ Based on interviews with DH managers/clinicians we assumed 40% of all referrals in DHs are surgical.^13^ To estimate the number of referrals in the Northern region we fitted a loglinear regression model using distance to referral center and district population as independent variables. Data on district population was derived from the 2018 Malawian census and distances were calculated using Google maps. When DHs were known to refer to multiple CHs (with some percentage) we used the weighted average as distance to referral center in the regression.

 The effectiveness of the intervention includes enhanced team work and increased capacity and confidence of DH-level surgical teams. We assumed that it takes time to build capacity and that without mentoring this capacity dissipates. In our models we therefore assume that the monthly number of referrals from a mentored DH follow an exponential decay function and that referrals return to normal with the same dynamics if there is no mentoring:


(7) $$\begin{array}{l} x\left(t\right)=\left\{\begin{array}{l} N_{0}e^{\frac{f\lambda t}{12}},\;if\;trip\;taking\;place\;in\;month\;t\\ N_{0}e^{\lambda t}\;if\;no\;trip\;taking\;place\;in\;month\;t\; \end{array}\right.\\ ,\;with\;t\leq t_{1}\;and\;x\left(t\right)\in \left[\frac{1}{3};1\right] \end{array}$$

Here, *N*_0_ is the effectiveness at the start of the intervention (it is 100% at the start, ie, no reduction in referrals), and *λ* the exponential decay constant. Parameter *λ* was estimated through a loglinear model based on referral data collected at QECH between 2018 and 2020.^11^ Note the inclusion of a $\frac{f}{12}$ term to model that the frequency of trips is assumed to influence the speed with which referrals from a mentored facility decrease. In another recent study we found that one third of incoming referrals at QECH were unnecessary, many of which could have been managed locally at the DH.^11^ We therefore assumed that no more than two-thirds of surgical referrals could be reduced. Based on the study by Mwapasa et al cited earlier the percentage of avoided referrals that underwent surgery locally was set at 25% and the percentage of avoided referrals that were managed conservatively was set at 10%.^7^ Trainer capacity at CHs was derived from an unpublished capacity assessment conducted in 2019 (P. Noah and E. Borgstein, unpublished data).We assumed the average length-of-stay of a referred surgical patient at a CH was 6 days.^11^

 The cost per referral for Nsanje, Mwanza and Mulanje (all in the Southern region) was obtained from an earlier study conducted in the SURG-Africa project.^13^ Where no empirical data were available, the cost per referral for DHs and the cost of a mentoring trip were imputed using a loglinear regression model that used distance to referral center and mentoring center. The marginal costs for each additional procedure, *c*_si_, was calculated from an earlier costing study.^14^ We adjusted the unit cost in that study by subtracting the capital costs and adjusting for case-mix to obtain the marginal recurrent costs. For hospitals where this study did not provide data, we imputed using the mean marginal recurrent cost. The costs of the intervention *c*_mi_ for hospitals in the Southern region was estimated using SURG-Africa budget and expenditure data. Data from periodic field trip reports and vehicle logs were used to differentiate between expected costs and actual expenditures. Expected fuel usage was set at 9 kilometers per 1 liter of fuel, with an extra 3-5 liters per trip depending on the distance to be covered. A fuel price of 850k MWK (Malawian Kwacha) per liter was assumed. For the expected cost per mentoring trip for DHs in central and northern regions we fitted a linear model using distance to mentoring facility as the sole predictor. As the project used government rates for the travel-subsistence allowance, we assumed these costs would not change if mentoring was taken up by the healthcare system. We assumed most costs associated with mentoring visits are variable, ie, that costs would be negligible when no visits occurred (eg, due to a cancellation).

 As some parameters are stochastic we ran each scenario 1000 times and used the mean model outcome.

###  Sensitivity Analyses

 To determine if our assumptions regarding certain key parameters might have influenced model outcomes, we ran univariate deterministic sensitivity analyses, involving 5 parameters: % avoided referrals managed conservatively (*β*), % costs incurred at ward (*α*), % avoided referrals operated locally (*γ*), % referrals that are surgical (*ρ*), and speed constant for mentoring effectiveness per trip (*λ*). The first four parameters are percentages, so we varied them from 0% to 100%. The fifth and last one (*λ*) is an exponential decay constant derived from a loglinear model based on referral data. We varied it from 0 (signifying that the intervention had no effect) to the lower bound of its 95% confidence interval (assuming a normally distributed error). The impact of these parameters was evaluated for the following outcomes: total referrals, total DH costs for surgery, total DH costs for surgical referrals, total mentoring costs, and total (extra) case load for CHs.

## Results

###  Group Model Building Results

**Figure 1:**
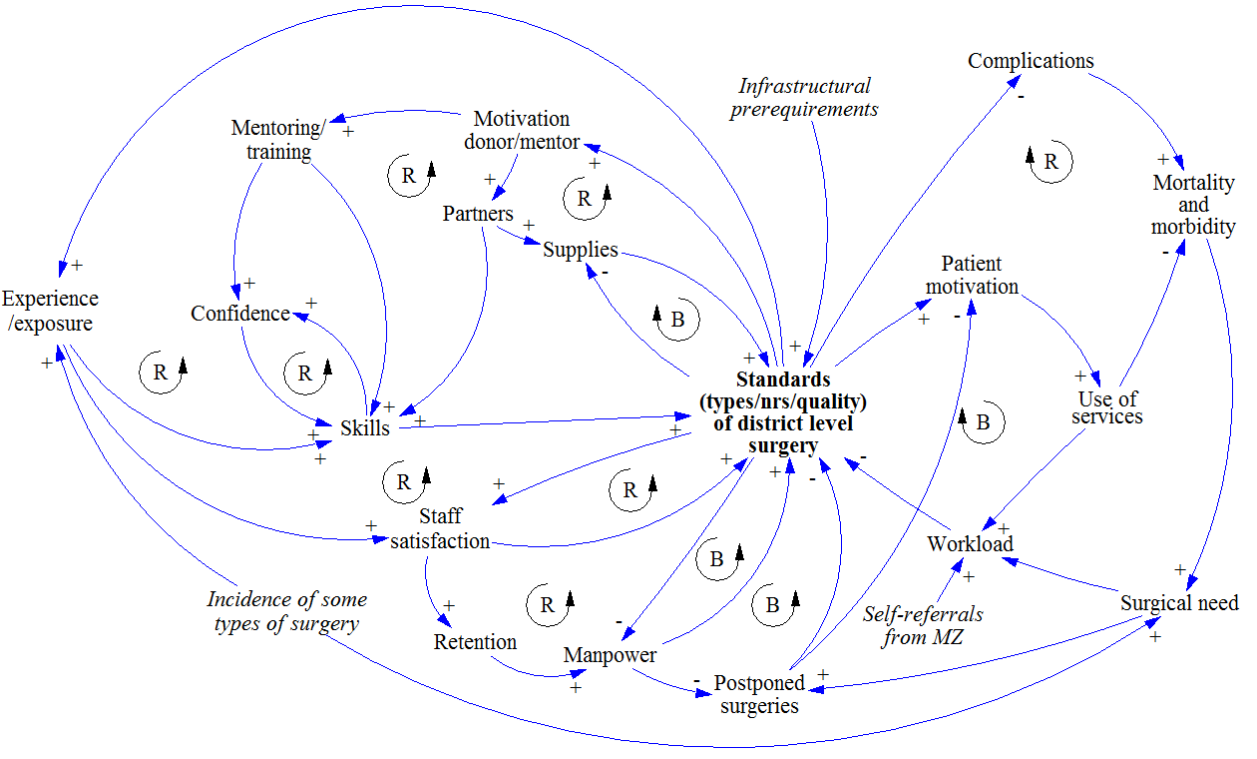
CLD Created in the Nsanje GMB Workshop. Arrows denote causal relationships as indicated by the workshop participants. A + indicates factors change in the same direction, a - indicates factors change in the opposite direction. ‘R’s denotes self-reinforcing feedback loops, while ‘B’ denotes balancing (ie, self-correcting) feedback loops. Factors in italic are not part of a feedback loop. Abbreviations: CLD, Causal loop diagram; GMB, group model building.

 The final CLD of the Nsanje GMB consists of 52 factors and has 12 feedback loops affecting the central variable, which was defined as ‘Standards (types/numbers/quality) of district level surgery’ (Figure 1).

 Although there are many interlinking factors, several themes emerge. First of all, the basic physical requirements for surgery are: supplies, equipment, infrastructure, and electricity. Secondly, GMB participants mentioned the importance of staff and their motivation (variables in green). A third theme is the motivation of patients (blue variables). All these themes affect and are affected by the standards of district level surgery. Finally, we see that several (long term) outcomes of improved standards of surgery are mentioned: reduced referrals to QECH, reduced morbidity and mortality, and an alleviation of surgical need. Some broad issues that affect many variables in the CLD were also mentioned: competing priorities for DHs, sources of finance for surgery, and budget ceilings. The CLD shows many self-reinforcing feedback loops, reflecting changes in surgical standards that can have long-term growth effects. Examples of this are a more surgically self-sufficient DH staff after they have been trained and mentored; increased interest of donors to contribute if standards go up. However, there are also self-correcting feedback loops that make it hard to achieve and retain gains, such as overloaded staff, inadequate infrastructure/supplies and reliance on external funding for supplies and training.

**Figure 2:**
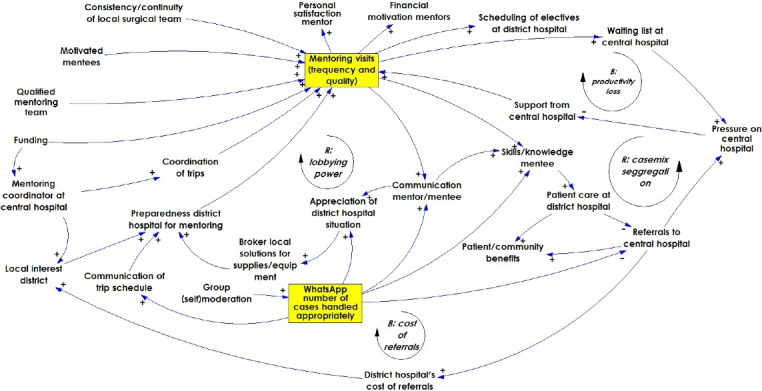
CLD created in the Salima GMB Workshop. The yellow factors represent the two components of the SURG-Africa surgical mentoring programme. Arrows denote causal relationships as indicated by the workshop participants. A plus sign (+) indicates factors that change in the same direction, a minus (-) indicates factors that change in the opposite direction. ‘R’ denotes self-reinforcing feedback loops, while ‘B’ denotes balancing (ie, self-correcting) feedback loops. Abbreviations: CLD, Causal loop diagram; GMB, group model building.

 The core of the CLD from the Salima workshop (Figure 2) consists of two central factors that describe the intervention: ‘mentoring visits (frequency and quality)’ and ‘number of cases handled appropriately.’ The rest of the CLD has 53 factors. The ‘mentoring visits’ factor has nine feedback loops affecting it, while the ‘WhatsApp’ factor has none. The main combined *effects* of the mentoring visits and the remote surgical consultation network are improved relations and communication between surgically active personnel in central and DHs, which in time could lead to improved care at DHs, and a subsequent reduction in surgical referrals from DHs to CHs. This would in the long run have financial implications for the DH. If the number of surgical referrals decreases, DHs will incur less costs for referrals but more costs to perform surgery themselves. The identified *requirements* for a successful intervention are: motivation of mentors and mentees, coordination, and funding of mentoring trips. These, in turn, result in several policy requirements for the mentoring intervention to be sustained. First of all, much of the (continued) effectiveness depends on good communication between CH-level mentors and DH-level mentees, and their respective hospital management teams.

###  Scenarios Description

 From the GMB sessions and further discussions among participants and project researchers, 6 possible scenarios were defined. Table 2 has the descriptions of these scenarios. In the first scenario, financial support for the mentoring trips (but not the remote surgical consultation network) ends in early 2020 (which is what actually happened). This is the comparator scenario as it entails no continuation of visits. In the second scenario, the intervention is sustained in the Southern region. In the third scenario the intervention is sustained in the Southern region and expanded to the Central region. The fifth scenario entails a nationwide roll-out and the sixth scenario is a nationwide roll-out with 6 instead of 4 visits per DH per year. Based on SURG-Africa schedule data we calculated that the probability of trip cancellation with 4 visits per year was 5% per visit. We assumed that in a scenario of intense mentoring (ie, 6 visits per DH per year) this probability would be 25%.

**Table 2:** Scenario Descriptions

**Scenario Name**	**Included Regions**				**Frequency of Mentoring in Years 3-5 (Trips Per DH Per Year)**	**Probability of Trip Cancellation**
	Year 1*	Year 2*	**Year 3**	**Year 4/5**		
Stop after SURG-Africa	S	S	-	-	-	-
Sustain in southern region	S	S	S	S	4	5%
Roll-out to central region	S	S	S+C	S+C	4	5%
Staggered roll-out	S	S	S+C	S+C+N	4	5%
Roll-out nationwide	S	S	S+C+N	S+C+N	4	5%
Roll-out nationwide (intense)	S	S	S+C+N	S+C+N	6	25%

Abbreviations: DH, district hospital; S, southern region; C, central region; N, northern region. Asterisks denote the SURG-Africa project years.

###  Resource Requirement Forecasts

**Figure 3:**
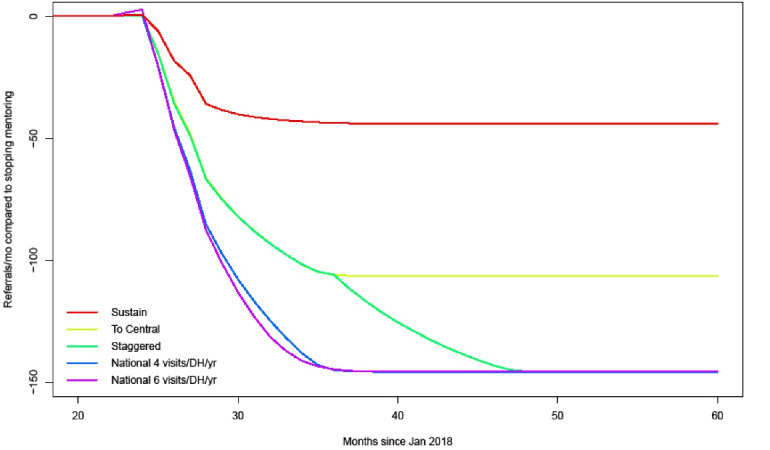
Referrals Per Month Across Malawi Compared to the Scenario of Stopping Mentoring at Month 24. Abbreviations: DH, district hospital; yr, year.

 The projections of referrals in the different scenarios are presented in Figure 3. The resource requirement forecasts that followed from these are presented in Table 3. In all 5 scenarios, referrals are expected to (further) decrease compared to the scenario of stopping surgical mentoring. The magnitude of decrease is larger when more regions are included in mentoring. The effects of trip cancellations are visible in this graph as temporary slower reductions in referrals. The staggered scenario departs from the ‘roll-out to Central’ scenario line at 36 months, when mentoring is expanded to the Northern region. The speed with which referrals decrease depends on the frequency of mentoring trips, the activity on the managed clinical network, and the probability of cancellations. Although we assumed the probability of cancelation for the intense national roll-out scenario was 5 times as high than the other scenarios, we still found that referrals in that scenario went down quickest. This makes sense as with a cancellation probability of 25% the expected number of visits per DH per year is 0.25 * 6 = 4.5, which is higher than the expected 0.95 * 4 = 3.8 in the other scenarios.

**Table 3:** Comparison of Resource Requirement Forecasts of Scenarios, Relative to the Scenario of Stopping Mentoring (Scenario 1)

		**Scenario 2: Sustain in Southern Region**	**Scenario 3: Roll-out to Central Region**	**Scenario 4: Staggered Roll-out**	**Scenario 5: National Roll-out**	**Scenario 6: Intense National Roll-out**
Change in costs (MWK per year)	… of mentoring	23M	46M	56M	61M	74M
	… of mentoring per avoided referral	1455k	3537k	4331k	4728k	4619k
	… of referrals	-25M	-56M	-76M	-88M	-89M
	… of surgery at DH	25M	60M	74M	82M	83M
Change in specialist days lost (per year per mentor)	QECH	42	42	42	42	49
	Kamuzu CH	-	34	34	34	41
	Mzuzu CH	-	-	13	19	23
Change in bed occupancy (bed days per year)	QECH	-2544	-2545	-2544	-2544	-2502
	Kamuzu CH	-	-3981	-3980	-3979	-4053
	Mzuzu CH	-	-	-1559	-2503	-2550
	Zomba CH	-406	-406	-406	-406	-412
Mentoring CH ‘return on investment’ (bed day reduction per mentoring day)	QECH	61	61	61	61	42
	Kamuzu CH	-	117	117	117	99
	Mzuzu CH	-	-	120	132	111

Abbreviations: QECH, Queen Elizabeth Central Hospital; DH, district hospital; CH, central hospital; MWK, Malawian Kwacha.

**Figure 4:**
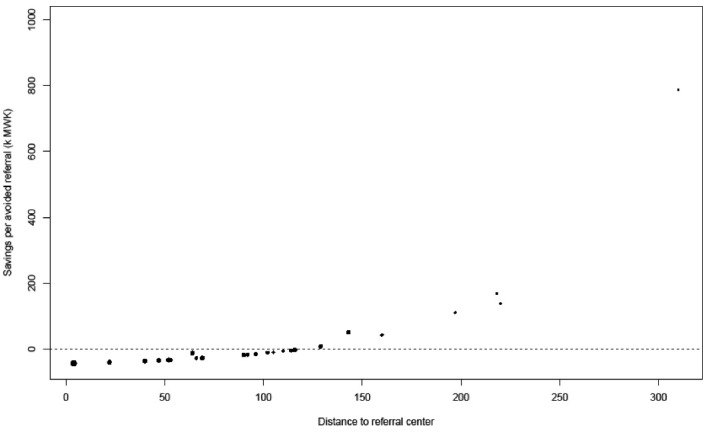
Potential Cost Savings Per Avoided Referral From the DH Perspective. This takes into account savings on referrals and extra costs for operating patients locally or managing them conservatively on the ward (see Formula 1). Abbreviation: DH, district hospital.

 The costs of mentoring are highest in the intense national roll-out scenario and lowest in the scenario where mentoring is only sustained in Southern region. The mentoring ‘investment’ per avoided referral goes up when the intervention is expanded to Central and Northern regions. From the DHs perspective, mentoring is cost-saving in 3 scenarios and it breaks even in other 2. There is a difference in the expected savings between hospitals: DHs that are farther away from their referral center are more likely to save money on avoided referrals (Figure 4). When their region is included, mentors at QECH, Kamuzu, and Mzuzu would need to devote about 42, 34, and 13 days to mentoring per year, respectively. When mentoring frequency is 6 visits per DH per year, this requirement would increase to 49, 41, and 23, respectively. In return, CHs would see a decrease in required bed-days; with this being more in the case of more intensive mentoring. The ‘return on investment’ for QECH, Kamuzu, and Mzuzu would be about 61, 117, and 132 bed days gained per mentoring day provided, respectively. This is lower in the 6-visit scenario because of the effect of cancellations: 42, 99, and 99. That the return on investment is lowest for QECH across all scenarios is because some of the referrals that are avoided by QECH mentors would otherwise have gone to Zomba CH. In most Zomba CH would save about 406 bed days without needing to provide mentors.

###  Mentoring Trip Coordination and Funding: A Question of Funds and Mentoring Capacity

 Participants of the Salima workshop considered the MoH the most appropriate party to fund and coordinate surgical mentoring. They recommended that the MoH establishes a focal person/desk that coordinates mentoring and other activities in support of district-level surgery. Inclusion of (surgical) mentoring as an activity in the annual District Implementation Plans, with a dedicated budget line, could be instrumental to sustain the mentoring program. Important areas for investment are in the training of adequate numbers of mentors, incentives for better staffing at the district level (ideally one clinical officer with a BSc in surgery), and adequate supplies for both mentoring and regular surgical duties. It was noted that a risk during SURG-Africa was that some DHs accumulated cases they are ideally expected to perform routinely on their own (or even cases that should otherwise be referred to the higher centers) with the expectation that the mentors would come and do them during the mentoring visit. In other cases there were not enough patients booked for the training. For these reasons, terms of reference should be developed for mentors and mentees that focus on good planning and make it clear that the visits aim to enable local teams to do more surgery through teaching and mentoring.

 It seems more cost-effective to have four visits per DH per year than 6. This is because in both cases referrals will decrease to the same level (in our models, the theoretical upper limit of 66% referral reduction), only at a slightly different speed. This comes at more costs and at a larger probability of cancellation. To reach the optimal reduction in referrals it is more important that mentoring is sustained over a long time than that it is rolled out quickly and intensely. It is also important that the managed clinical network remains functional, because mentors and mentees can use it to discuss cases.^6^ Although there are (almost) no financial costs to it, moderation is needed to ensure the forum remains functional. After SURG-Africa ends, coordination needs to be done by specially assigned staff. If coordination is lacking it may happen that trips are cancelled because DHs are ready or because visits are planned on days that do not align with mentor duties. A recommendation from GMB participants was that CH should employ a check list or a manual they can use before travelling to the DH to make sure that the DH is actually capable of receiving them in terms of available mentees and supplies.

 Whether or not cancellations affect the costs of the intervention depends mainly on how funds for mentoring are disbursed. If they are conditional on successful completion of field trips, cancellation of such trips may be harmful to the extent that they may induce inappropriate surgical referrals. If, on the other hand, funds for mentoring are disbursed regardless of successful completion of trips then there is a risk of dwindling motivation and wastage. In any case, trip cancellations would undermine learning effects and erode motivation of mentees, especially if DH staff have prepared themselves to receive and work with the mentors.

###  Maintaining Central Hospital Support When Benefits Are Not Immediately Visible

 Insufficient support on the side of CHs may be a risk. GMB participants mentioned that the absence of a specialist surgeon is immediately noticeable, while the benefits of fewer referrals are observed only after the mentees will have gained additional knowledge, skills and confidence. This has an opportunity cost and it may increase the workload on fellow specialist surgeons at the CH. On the other hand, in the long-term surgical mentoring might be expected to reduce the number of unnecessary surgical referrals and decongest the CH. The estimated resource requirements suggest that the trainer capacity required for mentoring would be substantial, especially in a scenario of nationwide roll-out with 6 trips per DH per year. This finding is in line with the Salima participants’ recommendations for CHs: focus on training and availability of mentors, and involve more specialties in the mentoring teams.

 In the resource requirement forecast models, the return on investments for CHs are substantial, but we calculated these numbers across the time horizon. From Figure 3 it appears that it can take a year or more before the maximum reduction in referrals is reached. It takes many mentoring visits to get there. This may make CHs, especially at the start, hesitant to send mentors even though in the long run the CH might benefit from fewer incoming referrals. Most surgical specialists have assigned theatre days, at QECH usually 2 days in a week. To minimize productivity losses at the CH, it is important that mentoring is well coordinated so that mentoring visits are planned on non-OT-days for the respective mentors. In addition, it is important that mentee motivation is maximized and mentee transfers to other facilities are minimized. Two factors that were not captured in the models was the quality of surgical referrals and the additional costs of surgery at the CH due to incoming referrals. The increase in quality of referrals as observed under SURG-Africa may be expected to increase CH motivation to participate in mentoring as the case mix shifts to more specialized and CH-appropriate surgery.^7^ As specialized surgery tends to be more expensive, costs are expected to go up when the number of unneeded referrals from DHs decreases.

###  Continuity and Motivation of Mentees

 For DHs, the main recommendation from the stakeholders in Salima was that DHs should try to ensure the availability of mentees and make a firm commitment to work with the mentors. The feasibility of these two recommendations is contingent on mentee motivation while it may be threatened by staff transfers. In the Salima GMB, mentee motivation was dependent on there being a mutual understanding between mentors and mentees that there was a fair share of benefits. It was mentioned that some mentees had complained that mentors received an allowance while they themselves did not. Mentors, on the other hand, argued that their allowance, based on government rates, was only to cover incurred expenses; and more importantly, that mentees would need to see the opportunity to increase their knowledge and skills as the main motivation to take part in the mentoring intervention. Staff transfers are a common occurrence in Malawi, both within and between hospitals. With changing local teams, mentors found it hard to establish relationships with mentees and mentor them. It was also mentioned that some DHs tended to save up complex surgical cases for the mentors to operate on during mentoring visits. Although patients benefited from this, it would undermine the mentoring’s main aim of strengthening local surgical capacity.

###  Difficulties of Scaling Up Surgery at DHs Under Severe Financial Constraints

**Figure 5:**
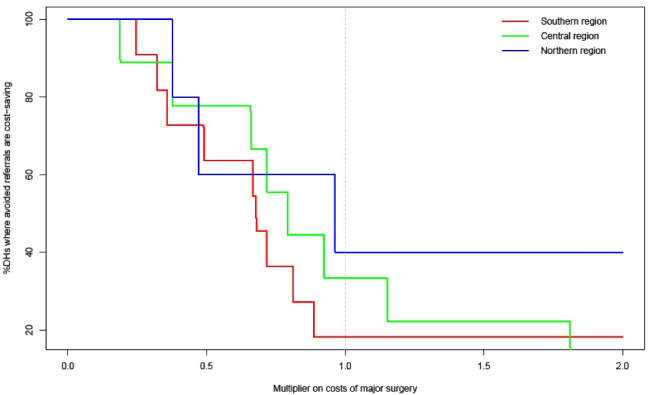
Threshold Analysis Showing the Proportion of DHs for Which an Avoided Referral Would Mean a Cost Saving (see Formula 1), as a function of a multiplier on the cost of major surgery. The proportion of DHs for which avoided referrals imply cost savings goes up when our estimate for the costs of major surgery goes down. Abbreviation: DH, district hospital.

 From earlier work we know that the financial situation at many Malawian DHs is dire, with many being dependent on donor funds and in debt with local suppliers. A major risk to the sustainability of the mentoring intervention is therefore the DHs’ financial capacity to undertake surgery on patients that they would no longer refer after having increased their technical capacity as a result of mentoring. Our resource estimates suggest that for many DHs (especially those closer to referral centers), the financial savings of fewer referrals may be about equal or less than the additional costs they would incur from performing more surgery locally (Figure 4). For about 50%, avoided referrals do not imply cost savings. A risk of this is that even if local team skills improve other considerations such as a lack of supplies or properly maintained equipment may prohibit them from undertaking certain surgical cases at the DH level. If they do perform the surgery locally, there is a risk of other priority activities at the DH receiving less funding. The balance hinges on the uncertainty in our estimate for the cost of a major surgery at the DH level. Figure 5 shows a threshold analysis of how the percentage of DHs for which an avoided referral would mean a cost saving as a function of this parameter. This shows that if we overestimated the cost of DH level surgery, the probability of savings increases. If our estimate turns out too low, then our results would not change much.

###  Results of Sensitivity Analyses

 Whether or not the intervention is cost saving from a DH perspective hinges on the uncertainty in our estimate of the cost of a major surgery at the DH level. Figure 5 shows a threshold analysis of the percentage of DHs for which an avoided referral would mean a cost saving, as a function of this parameter. This shows that if the cost of surgery at the DH is overestimated, the probability of district-level savings increases. If the estimate turns out too low, then the results would not change much.

 Supplementary files 2 to 6 present graphs produced in the sensitivity analyses that show the effects of different parameter values on five outcomes. Overall the model outcomes are most sensitive to the estimates of mentoring effectiveness and the percentage of referrals labeled as surgical. It appears there is little room for improvement on just the intervention side (Figure A5). This figure shows that a lower level of effectiveness implies more surgical referrals. Although higher effectiveness (relative to our estimate) would further lower surgical referrals, the effect is small. All parameters that influence referrals automatically have an impact on the costs of referrals, the costs of surgery, and the work load of CHs; and this is proportional to the change in referrals (Figures A10, A15, A25). Regarding impact of the percentage of surgical referrals on total referrals, Figure A4 shows that with a lower percentage of patients requiring surgical care, total referrals go up (because the intervention is assumed affect only surgical patients). Another important observation is that the costs of mentoring remain the same, regardless of its effectiveness (Figure A20); it means that the intervention would be less cost-effective, as fewer referrals are avoided for the same investment.

## Discussion

 In this study we investigated issues surrounding policy adoption of a surgical mentoring intervention in Malawi. We used both qualitative stakeholder input and quantitative modeling to inform possible scenarios for sustaining and rolling out surgical mentoring. Policy adoption and the articulation of new mandates in a complex adaptive system such as the healthcare system in a resource-constrained environment is a topic of much recent debate. In a commentary on the role of complexity in health services research, Greenhalgh notes that “*the articulations, workarounds and muddling through that keep the show on the road are not footnotes in the story, but its central plot.*”^15^ For sustainable policy, it is often not wise to rely on completely articulated ‘sustainment plans’ that spell out actions for the coming years because the potential outcomes of complex systems are hard to predict. The healthcare system is composed of many actors, each with their own perspective and sphere of influence. Chandler argues that many rules guide the behavior of a healthcare system change over time and that these are hard to identify.^16^ This makes intervening hard and likely to have unintended consequences. She argues that instead of creating new rules in the system through intervention (plans), implementers instead should focus on finding ways to incentivize actors so that new (beneficial) behavior emerges. From our study we conclude that there is a strong case for the MoH in Malawi to work with its CHs towards institutionalization of district-level surgical team mentoring. Although the system-wide benefits of such mentoring are acknowledged by all stakeholders, the incentives may not be directly apparent or even be insufficient, which would preclude a nation-wide roll-out. When surgical specialists engage in mentoring activities, CHs may immediately feel the loss of specialist time, with the benefit of reduced referrals coming only later. DHs may not be incentivized to do more surgery (even if their teams become more skilled/confident) if the costs of doing surgery locally exceed the savings gained from referring fewer patients, and especially if the government does not compensate DHs for these additional costs.

 We further argue that for the institutionalization of surgical mentoring a staggered nationwide roll-out is most suitable. Although the forecast model suggests that a staggered roll-out would be slower to yield benefits than an immediate national roll-out, it provides more time to develop, test and learn, and to implement coordination and monitoring mechanisms for sustainable surgical team mentoring. For the development of such mechanisms several recommendations can be made:

- Coordination is best done by a dedicated focal point in the MoH with specially assigned senior staff who are in regular contact with both CHs and DHs. As cancellations of mentoring trips may have negative effects (referrals can go back up, erosion of both CH and DH support), it is important to ensure DHs are ready to receive the mentors (eg, by prior telecommunication based on a standard checklist) and that cancellation due to conflicting mentor or mentee duties is minimized.
- Disbursement of travel-subsistence allowances is best made conditional on trip completion and delivery of trip reports. Such reports can serve as a tool for monitoring compliance to agreed mentoring procedures. If reports are incomplete, submitted late or filled out by junior staff, this may be a sign of eroding compliance.
- It may be beneficial to include in-service training and mentoring in the key performance indicators for both DHs and surgical specialists for motivation purposes.

In a continuation of participatory action research work done under SURG-Africa,^9^ it was found critical for mentors and mentees to jointly finetune the intervention through periodic meetings, to improve both its acceptability and effectiveness.

 If surgical mentoring will actually result in more DH-level surgery, there may be a need to coordinate (more) closely with district health administrators (including those who are in charge of personnel, supplies and finance) so as to secure adequate resources.

 A system-wide issue that may undermine the sustainability of mentoring, even if it is well-coordinated, is the financial situation of DHs. DHs in Malawi provide most services (including hospital admission and surgery) free of charge despite the costs they incur for supplies, repairs, utilities, and kitchen services. In our models about 50% of DHs would incur losses for each avoided referral, especially those close to referral centers. This may discourage them from scaling up surgery unless they are compensated somehow for the surgical services they provide. Surgery not being a national programme in Malawi and not having a dedicated budget line, there is no such compensation. We would therefore argue for a kind of output-based or performance-based resource allocation: the more surgery a hospital provides, the more (government) budget they would be entitled to.

 The major strength of this study is that it combined stakeholder input with evidence and dynamic models to explore policy options. The ideal circumstances of evidence-based policy are, like in many real-world instances, not present in this study. This means that we had to make do with best available evidence and/or make assumptions based on expert input. The question is, of course: was there any alternative? Nation-wide randomized controlled trials of surgical mentoring policies are not feasible. We would argue that results from a mixed-method approach such as the one employed here are valuable for policy exploration as long as they are not taken as absolute evidence for future planning and budgeting, but rather as an entry point for further improvements and roll-out.

 The main limitation of this study is that for the post-2020 forecasts we assumed other major system factors to remain constant, such as the need for surgery and the number of available mentors. A pointed and unanticipated example of this limitation is the COVID-19 pandemic, which is having a major effect on Malawi’s healthcare system but was not considered at the GMB workshops. A second limitation is that the models used data and perspectives from the Southern region mostly. It is likely that our forecasts for the Central and Northern regions would have been different, had we had access to empirical data from those regions. Another limitation is that we did not take surgical referrals from health centers (rather than from DH) or inter-CH referrals into account in the quantitative models. Finally, we did not take into account any residual effects from the remote surgical consultation network which is likely to continue under all scenarios. As such, we expect it would affect all scenarios equally and not significantly impact our findings.

## Conclusion

 This paper explored the complex dimensions of implementing surgical team mentoring. Although there are tangible system-wide benefits, including benefits for patients, the complexity of the healthcare system in Malawi makes sustaining and rolling out surgical mentoring a non-straightforward affair. To institutionalize surgical mentoring, it is important to take an approach of staggered roll-out, coordination, provision of appropriate incentives, and periodic monitoring. In particular, an output- or performance-based financing scheme for DHs would be required to incentivize them to scale up surgery.

## Acknowledgements

 We thank the GMB workshop participants for their valuable contributions. We are thankful for the assistance of Dr. Monic Lansu of Radboud University in preparing the GMB workshops.

## Ethical issues

 Ethical approval was granted by the College of Medicine Research and Ethics Committee (COMREC) on February 14, 2018 (P.01/18/2336).

## Competing interests

 Authors declare that they have no competing interests.

## Authors’ contributions

 Conception and design: HB, LB. Acquisition of data: HB, MI, GM, CP, PN, JG, and LB. Analysis and interpretation of data: HB, MI, and LB. Drafting of the manuscript: HB. Critical revision of the manuscript for important intellectual content: all authors. Statistical analysis: HB. Obtaining funding: NM, EB, RB, JG, and LB. Supervision: LB.

## Funding

 This work was performed under the SURG-Africa project, funded by the European Union’s Horizon 2020 Programme for Research and Innovation, under grant agreement no. 733391.

## Supplementary files


Supplementary file 1. List of 23 Government-Owned District Hospitals.

Supplementary file 2. Sensitivity Analysis of Parameter Effects on Total Referrals (Figures A1-A5).

Supplementary file 3. Sensitivity Analysis of Parameter Effects on Costs of Referrals for District Hospitals (Figures A6-A10).

Supplementary file 4. Sensitivity Analysis of Parameter Effects on Cost of Surgery for District Hospitals (Figures A11-A15).

Supplementary file 5. Sensitivity Analysis of Parameter Effects on Total Mentoring Costs (Figures A16-A20).

Supplementary file 6. Sensitivity Analysis of Parameter Effects on Total Case Load at Central Hospitals (Figures A21-A25).
