# Supplementary material for: Improving Access to Surgery Through Surgical Team Mentoring – Policy Lessons From Group Model Building With Local Stakeholders in Malawi
Source: Int J Health Policy Manag. 2021 Aug 3;11(9):1744–55. doi: 10.34172/ijhpm.2021.78 (PMC9808242; doi:10.34172/ijhpm.2021.78)
Supplement: Supplementary file 1 — List of 23 Government-Owned District Hospitals. [file ijhpm-11-1744-s001.pdf]

Article title: Improving Access to Surgery Through Surgical Team Mentoring – Policy Lessons From Group Model Building With Local Stakeholders in Malawi

Journal name: International Journal of Health Policy and Management (IJHPM)

Authors' information: Henk Broekhuizen[1,2], Martilord Ifeanyichi[1], Gerald Mwapasa[3], Chiara Pittalis[4], Patrick Noah[3], Nyengo Mkandawire[3], Eric Borgstein[3], Ruairí Brugha[5], Jakub Gajewski[4], Leon Bijlmakers[1]*

[1]Radboud Institute for Health Sciences, Radboud University Medical Centre, Nijmegen, The Netherlands.

[2]Department of Health and Society, Wageningen University and Research, Wageningen, The Netherlands.

[3]College of Medicine, Blantyre, Malawi.

[4]Institute of Global Surgery, Royal College of Surgeons in Ireland, Dublin, Ireland.

[5]Department of Epidemiology and Public Health Medicine, Royal College of Surgeons in Ireland, Dublin, Ireland.

(*Corresponding author: Leon.Bijlmakers@radboudumc.nl)

Supplementary file 1. List of 23 government-owned district hospitals

Source data used in resource requirement calculations. Single asterisks (*) denote empirical data from SURG-Africa; double asterisks (**) denote values from literature; other values were imputed using (log)linear regression models.

DH=district hospital, C=Central region, N=Northern region, S=Southern region, QECH=Queen Elizabeth Central Hospital, MWK=Malawian Kwacha

					Cost per …			Referring patients to …			
**District hospital**	**Region**	**Weighted distance (km)**	**Referrals 2018**	**District population****	**Mentor trip (1000 MWK)**	**Referral (1000 MWK)**	**Major surgery (1000 MWK)**	**Mzuzu CH**	**Kamuzu CH**	**QECH**	**Zomba CH**
Bwaila	C	4	171**	989,318	341	10	184	-	100%	-	-
Dedza	C	90	114**	830,512	616	35	184	-	100%	-	-
Dowa	C	52	794**	772,569	495	20	184	-	100%	-	-
Kasungu	C	129	120**	842,953	741	61	184	-	100%	-	-
Mchinji	C	114	86**	602,305	693	49	184	-	100%	-	-
Nkhokota	C	197	257**	395,897	958	164	184	-	100%	-	-
Ntcheu	C	160	129**	659,608	840	96	184	-	100%	-	-
Ntchisi	C	96	544**	317,069	635	38	184	-	100%	-	-
Salima	C	102	193**	478,346	655	42	184	-	100%	-	-
Chitipa	N	310	86	234,927	1320	841	184	100%	-	-	-
Karonga	N	218	86	365,028	1026	222	184	100%	-	-	-
Mzimba	N	116	103	936,250	699	51	184	100%	-	-	-
Nkhata Bay	N	53	616	285,795	498	20	184	100%	-	-	-
Rumphi	N	66	574	229,161	539	25	184	100%	-	-	-
Mangochi	S	143	229*	1,148,611	786*	75	83**	-	-	25%	75%
Mulanje	S	64	355*	684,107	533*	24*	125**	-	-	100%	-
Nsanje	S	220	286*	299,168	1032*	229*	314**	-	-	100%	-
Chikwawa	S	47	416*	564,684	479*	19	184	-	-	100%	-
Chiradzulu	S	22	342*	356,875	399*	13	184	-	-	100%	-
Machinga	S	69	207*	735,438	549*	26	184	-	-	25%	75%
Mwanza	S	105	166*	130,949	664*	43*	184	-	-	100%	-
Phalombe	S	92	171*	429,450	623*	36	184	-	-	100%	-
Thyolo	S	40	423*	721,456	456*	17	184	-	-	100%	-
