# Supplementary material for: Improving Access to Surgery Through Surgical Team Mentoring – Policy Lessons From Group Model Building With Local Stakeholders in Malawi
Source: Int J Health Policy Manag. 2021 Aug 3;11(9):1744–55. doi: 10.34172/ijhpm.2021.78 (PMC9808242; doi:10.34172/ijhpm.2021.78)

Article title: Improving Access to Surgery Through Surgical Team Mentoring – Policy Lessons From Group Model Building With Local Stakeholders in Malawi

Journal name: International Journal of Health Policy and Management (IJHPM)

Authors' information: Henk Broekhuizen[1,2], Martilord Ifeanyichi[1], Gerald Mwapasa[3], Chiara Pittalis[4], Patrick Noah[3], Nyengo Mkandawire[3], Eric Borgstein[3], Ruairí Brugha[5], Jakub Gajewski[4], Leon Bijlmakers[1]*

[1]Radboud Institute for Health Sciences, Radboud University Medical Centre, Nijmegen, The Netherlands.

[2]Department of Health and Society, Wageningen University and Research, Wageningen, The Netherlands.

[3]College of Medicine, Blantyre, Malawi.

[4]Institute of Global Surgery, Royal College of Surgeons in Ireland, Dublin, Ireland.

[5]Department of Epidemiology and Public Health Medicine, Royal College of Surgeons in Ireland, Dublin, Ireland.

(*Corresponding author: Leon.Bijlmakers@radboudumc.nl)

Supplementary file 5. Sensitivity analysis of parameter effects on total mentoring costs (Figures A16-A20)

Figure A16: Impact of percentage_avoided_that_conservative on total_mentoring_costs

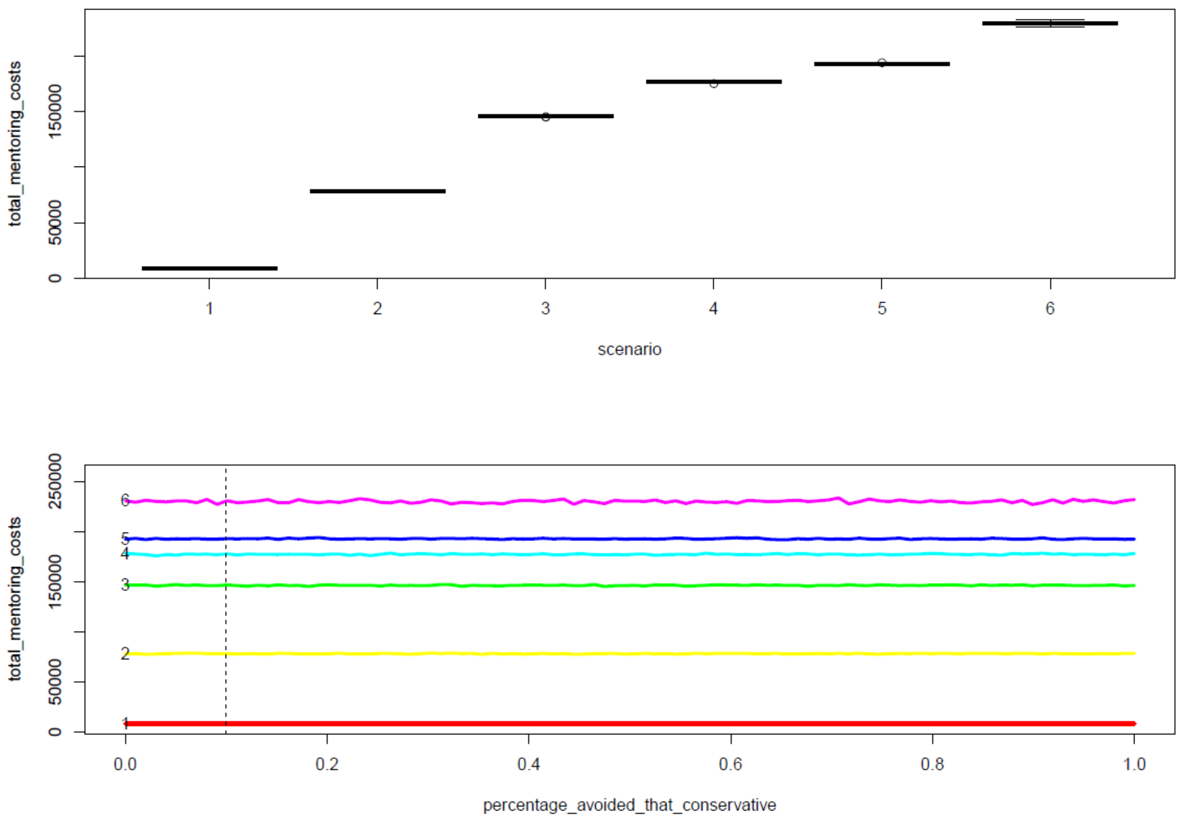

Figure A17: Impact of percentage_costs_ward on total_mentoring_costs

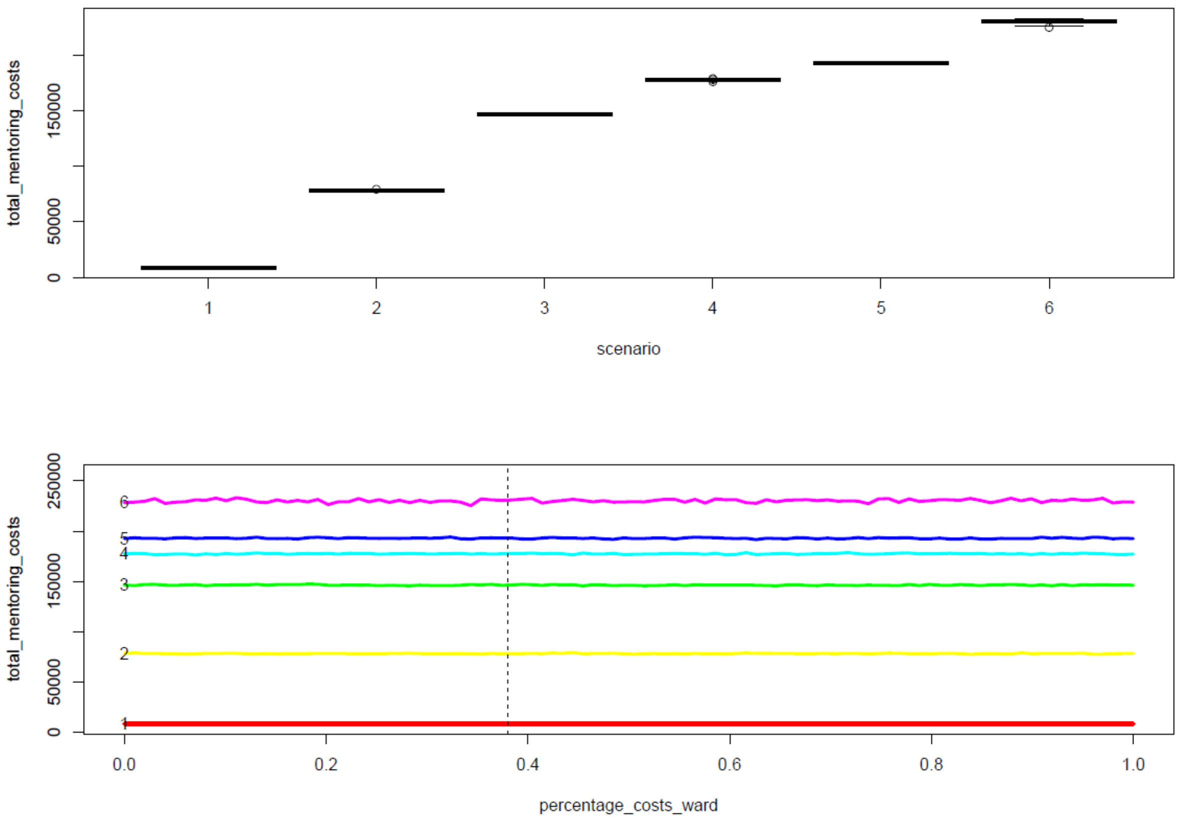

Figure A18: Impact of percentage_avoided_operated_locally on total_mentoring_costs

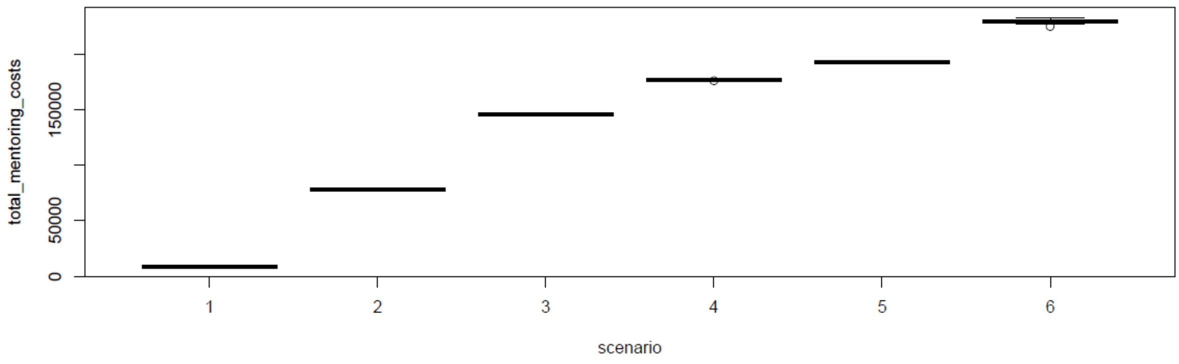

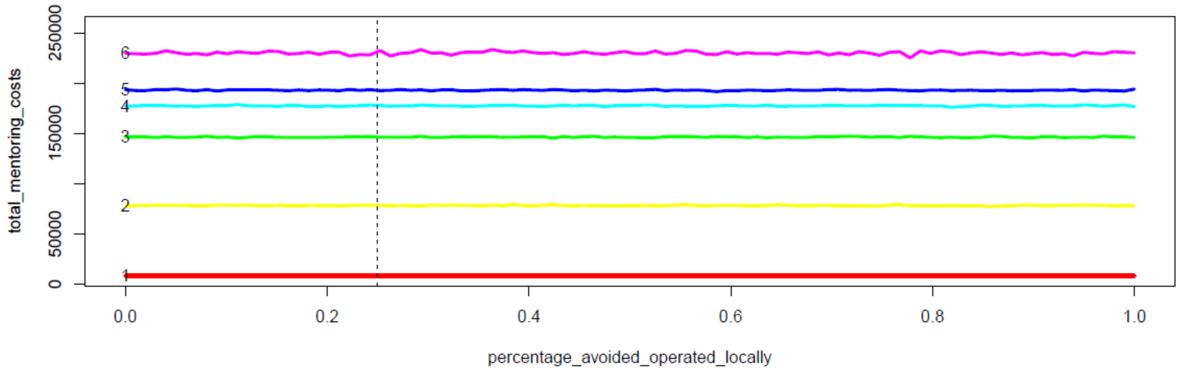

Figure A19: Impact of percentage_referrals_surgical on total_mentoring_costs

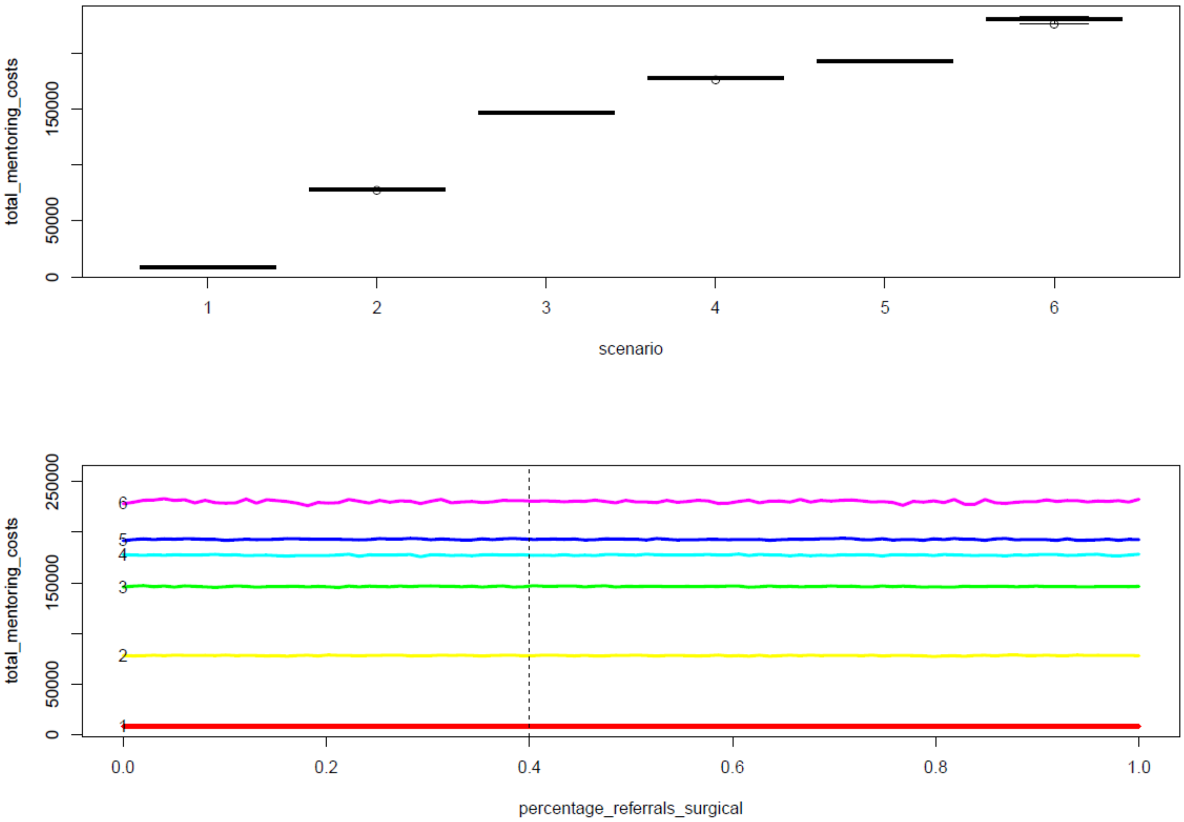

Figure A20: Impact of effectiveness_constant on total_mentoring_costs

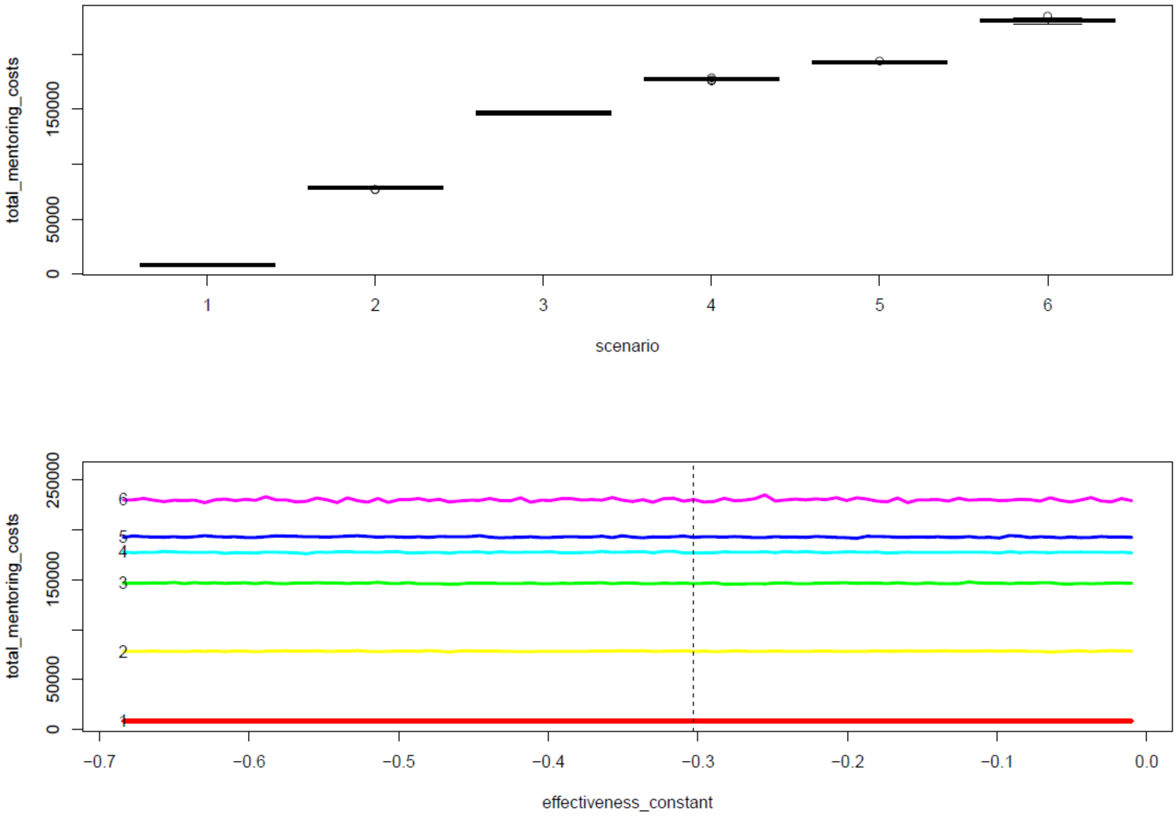

Supplement: Supplementary file 5 — Sensitivity Analysis of Parameter Effects on Total Mentoring Costs (Figures A16-A20). [file ijhpm-11-1744-s005.pdf]
